# SARS-CoV-2: Remarks on the COVID-19 Pandemic

**DOI:** 10.1007/s00005-020-00600-7

**Published:** 2020-11-13

**Authors:** Egbert Piasecki

**Affiliations:** Laboratory of Virology, Hirszfeld Institute of Immunology and Experimental Therapy, Polish Academy of Sciences, Rudolfa Weigla 12, 53-114 Wrocław, Poland

**Keywords:** COVID-19, SARS-CoV-2, Virus, Pandemic

## Abstract

The COVID-19 pandemic developing rapidly in 2020 is triggered by the emergence of a new human virus—SARS-CoV-2. The emergence of a new virus is not an unexpected phenomenon and has been predicted for many years. Since the virus has spread all over the world, it will be very difficult or even impossible to eradicate it. A necessary condition for complete or partial elimination of the virus is to have an effective vaccine. It is possible that SARS-CoV-2 will become milder in the next few years and COVID-19 will then only threaten individuals from risk groups.

## Introduction

The appearance of a new human virus is an interesting but not surprising phenomenon. Many predictions and scenarios of such an event have been published in recent years. They mainly referred to the expected influenza pandemic, but coronaviruses were also taken into consideration. It was also predicted that the next pandemic might be caused by a pathogen of unknown origin and taxonomic affiliation. The emergence of such a virus in the near future would result in the Disease X pandemic (Simpson et al. 2020). This hypothetical pandemic has now become real with the spread of SARS-CoV-2 around the world.

## The Origin of New Human Viruses

Human viruses could have arisen in two ways. First, there could be a co-evolution of the host (*Homo sapiens*) and its viruses. Mutual adaptation leading to the establishment of viruses with low pathological potential is most likely. As a consequence of this balance between host and virus, mostly asymptomatic cases of infection can be observed.

The second way is to introduce an animal virus into the human species. The sources of such zoonotic transmissions are mainly mammals, less frequently birds. The introduction of viruses adapted to replication in cells of less related species is less likely due to significant differences in host cell metabolism.

Humans were probably introduced to the most clinically important viruses relatively recently in the history of *Homo sapiens* evolution.

Descriptions of smallpox cases can be found in ancient documents written e.g. in Egypt, Mesopotamia, and Greece. However, Native Americans suffered from variola virus only after the arrival of Europeans in the Columbus era. The virus was new and deadly for them, far more dangerous than it was for the people of the Old World. This suggests that an animal poxvirus was introduced to the human population somewhere in the Old World, probably in Africa, not long before 2000 BC. A similar fatal effect on Native Americans was seen with the transfer of another European virus—the measles virus.

The human measles virus probably originated from a related paramyxovirus. Namely, the rinderpest (bovine measles) virus is believed to be the ancestor of the human virus. Divergence of the two viruses has been estimated between the eleventh to the twelfth century AD (Furuse et al. 2010), or, as recently published analysis indicates, the sixth century BC (Düx et al. 2020).

The natural history of new viruses can be described more precisely in contemporary times. Two human immunodeficiency viruses (HIV-1 and HIV-2) are believed to be the result of the introductions of simian viruses in the first half of the twentieth century. Chimpanzee and/or gorilla simian immunodeficiency viruses (SIV_cpz_ and SIV_gor_) are considered the ancestors of HIV-1. HIV-2, in turn, are probably derived from SIV_smm_ (SIV of sooty mangabey) (Reeves and Doms 2002; Santiago et al. 2002).

SIVs have been found in many African primates. The viruses usually cause asymptomatic infections. Only SIV_cpz_ can cause symptoms of immunodeficiency as a result of chimpanzee infection. However, the disease in chimpanzees is generally milder than human AIDS (Rutjens et al. 2003). These observations would support the hypothesis that old viruses become benign; however, it is difficult to compare the pathogenicity of viruses in different hosts. HIV is known to be a new virus; SIV_cpz_ is likely the result of a slightly older introduction, and other SIV viruses have been considered endemic for a long time. Moreover, Asian macaques lacking natural SIV infections are susceptible to infection with African monkey viruses and develop simian AIDS when infected (Chahroudi et al. 2012).

We currently know seven human coronaviruses. Four of them usually cause mild colds and infect humans for a long time (common cold coronaviruses—HCoV-OC43, HCoV-NL63, HCoV-229E, and HCoV-HKU1) (Cui et al. 2019; Pyrć 2015). Three new human coronaviruses originated in the twenty-first century. The first was the Severe Acute Respiratory Syndrome Virus (SARS-CoV, now called SARS-CoV-1) in 2002, the next—Middle East Respiratory Syndrome Virus (MERS-CoV) in 2012, and the latest is the causer of the current pandemic—SARS-CoV-2—found in 2019. Viruses found in different species of insectivorous bats are considered the ancestors of these new viruses (Cui et al. 2019).

A separate problem is zoonotic infection, where exposure to a virus specific for another species occurs incidentally. In most cases, such exposure does not lead to the development of an infection, as animal viruses are usually unable to replicate effectively in human cells. In other instances, zoonotic viruses typically produce only symptoms in an infected person with no or negligible transmission between people, e.g. rabies virus or H5N1 avian influenza virus.

What are the mechanisms underlying a new human viruses origin? What is the reason why a virus that has replicated so far in the cells of one species becomes able to replicate in the cells of another? If we exclude any directed action, the only possibility is random mutation or recombination. After such a change, the virus can infect a new host if such a host is available at the location of the virus. Therefore, contact with animals (primarily mammals) is potentially dangerous.

In the case of SARS-CoV-2, it is assumed that the bat virus has directly or indirectly become the human virus, maybe after recombination with a coronavirus of another mammalian species (Chan et al. 2020; Lu et al. 2020; Wu et al. 2020; Zhu et al. 2020). Novel coronavirus was established as a human virus when the animal shedding the altered virus was present near humans, probably somewhere in China.

The hypotheses that humans contributed to the development of SARS-CoV-2 while working on coronaviruses in scientific laboratories are currently unverifiable.

## Is SARS-CoV-2 Eradication Possible?

While we observe the emergence of new viruses attacking humans or other hosts, the reverse is exceptional. Typically, infections due to a new virus appear more or less frequently, and it cannot be hoped that they will clear up on their own.

Well-known examples of virus disappearances can be considered in three types of action:

### Virus Eradication

It has been successful in very few cases. In 1979, the eradication of the smallpox virus was announced (Henderson 1987). Bovine rinderpest virus as the second virus was eradicated in 2011 (Orzechowska et al. 2018; Roeder 2011). The eradication of polioviruses (PVs) is currently underway. It seems that two of the three species of poliovirus have been eradicated (PV2 in 1999 and PV3 in 2012). The remaining type 1 poliovirus turned out to be so difficult to eliminate that the eradication has not been progressing for several years. Moreover, problems with the circulation of attenuated viruses contained in live vaccines are increasing (Global Polio Eradication Initiative 2020).

In all of the above cases, the basis and condition for effective action is acquiring an effective vaccine that gives long-term immunity. The properties of eradicated viruses are also crucial. Smallpox virus and polioviruses are human viruses without a natural animal reservoir. Moreover, there are no problems with persistent infection cases.

The eradication plans for viruses such as measles virus, rubella virus, hepatitis B virus (HBV) and human papillomavirus type 16 (HPV-16) are currently under consideration. In all of these cases, we have an effective vaccine and there are no animal reservoirs of the viruses. However, chronic infections occur with HBV and HPV-16. Vertical transmission for HBV also makes the eradication plan difficult to follow. Of course, high financial costs are also involved.

Without an effective vaccine, it is impossible to imagine eradicating any virus. In the current COVID-19 pandemic, only when we have hundreds of millions of doses of the vaccine we will be able to consider the feasibility of SARS-CoV-2 eradication.

### Suppression of a New Infection

This was the case with SARS-CoV-1. The nature of the infection made it possible to isolate all infected persons, and the lack of chronic infections allowed us to eliminate the virus from the human population. However, it should be noted that this was an exceptional situation, which is unlikely to be repeated.

Nowadays, the course of SARS-CoV-2 infection (infectivity before the onset of symptoms, high percentage of asymptomatic cases) made such a scenario impossible to implement.

One may wonder if a drastic lockdown had been introduced in Wuhan at the beginning of December 2019, the virus could have been stopped. However, it does not seem likely. Looking at how other countries dealt with the pandemic, one can see that there is a predominantly defensive strategy. Only those countries that have adopted an offensive strategy based on mass testing have been partially successful. However, this strategy could not be used from the very beginning, when we had not yet recognized the virus, so there was no way to test.

### Spontaneous Disappearance of a Virus

It is not excluded that viruses can stop infecting humans without conscious human action. However, one should not count on it too much, although many historical sources say that “the plague came and went”. Unfortunately, usually, or perhaps always, the plague would go away only for a while.

Assuming actual virus elimination, mechanisms on the host side and on the virus side may be considered.

Such a virus elimination mechanism could be genetic selection leading to the population becoming insensitive to infection. This may have happened in the past, at times for which we do not have data, but is unlikely today. The reason is the lack of selection mechanisms leading to an increase in the frequency of the resistance genes in the population.

It is known that having certain alleles reduces the possibility of infection with a given virus. The best example is having a gene for inactive CC chemokine receptor type 5 (CCR5) in a homozygous state. The *CCR5-Δ32* homozygous individuals are practically resistant to HIV-1 infection. People with such a genotype are most common in Northern Europe (up to several per cent), less often in Central Europe (about 1% in Poland), and rare or absent in other regions of the world (Libert et al. 1998; Martinson et al. 1997; Zwolińska et al. 2013). Such geographical diversity probably results from the action of a selection factor in the past, but currently, such mechanisms are unlikely to work. On the other hand, it should be mention that often acquiring resistance to a given pathogen is associated with some dysfunction. In the case of CCR5, the lack of its functional version is associated with increased sensitivity to flaviviral diseases such as West Nile virus infection and tick-borne encephalitis (reviewed in Ellwanger et al. 2020).

In the case of SARS-CoV-2, it is not excluded that part of the population is insensitive and more likely some individuals are less susceptible to infection due to genetic reasons. However, the spread of the virus shows that these factors seem to be of little importance considering the development of the pandemic. Genetic factors suspected of affecting the severity of COVID-19 identified so far do not appear to have a strong effect, which can indicate a group of people resistant to the infection. However, it should be noted that the search for genetic factors is still poorly documented and is focused mainly on factors favoring the severity of the infection (Ellinghaus et al. 2020; van der Made et al. 2020; Zhang et al. 2020).

It does not seem possible for a virus to spontaneously change its properties and stop infecting its host without external causes. The observed changes in influenza virus strains do not contradict this thesis. Influenza A/H1N1 virus ceased to circulate in 1957, being replaced by A/H2N2, which in turn was replaced by A/H3N2 in 1968. At a lower level of differentiation, antigenically distinct strains of influenza viruses appear every few years. However, these changes are forced by an increase in the number of people who have acquired long-term specific immunity as a result of being infected with hitherto circulating strains.

The acquired immunity in the case of common cold coronavirus infections has proved to be short-lived. In the case of SARS-CoV-2 the situation will most likely be similar, which means that in the long term there will be no selective pressure on the virus towards major changes in its structure.

## What is the Threat? Will the Virus Become Milder?

In the face of the emergence of a new virus, the question arises: will the natural ability of the virus to mutate change the pathogenic properties of the virus, and finally will it become more or less virulent? In the case of SARS-CoV-2, we do not know yet. So far, it has been observed that it changes slowly. From an evolutionary point of view, a shift towards attenuation can be expected as such viruses can usually spread better. Viruses that produce less uncomfortable symptoms have an evolutionary advantage as long as it is not accompanied by a reduced possibility of spread and infection. This was the case with the Spanish influenza virus, which for two seasons 1918/1919 and 1919/1920 was particularly deadly, and then its milder descendants dominated. Coronaviruses are more genetically stable than influenza viruses and it will take more time, if any, for the virus to evolve to the milder forms (Ferron et al. 2018).

When animal viruses interact with their host, a simultaneous evolution of both the virus and the host may be observed. The example described was the story of rabbits and the myxomavirus. In the 1950s, Australia tried to exterminate rabbits by spreading the myxomavirus, which killed 99% of animals. The goal was not met, and after 10 years, the rabbits were again numerous and have become less sensitive to the virus that on its part has become milder (Angulo and Cooke 2002). In the case of humans, perhaps such processes took place in the past and we have adapted genetically to new viruses that were dangerous at the beginning. Currently, however, there is no such selection pressure. In addition, the speed of consolidation of genetic changes is obviously conditioned by generation time, which is relatively long in human population, calculated for more than 20 years.

Typically, the evolutionary success of a virus is associated with the lack of significant host pathology. However, it does not always have to be this way. In some instances, the increased pathogenicity may also occur and lead to better spread of a virus. The sequence analysis of the smallpox virus samples derived from ancient times indicated that around the sixth century AD the virus became more virulent due to the loss of certain genes and kept such properties until successful eradication of the virus in twentieth century (Mühlemann et al. 2020).

The observed SARS-CoV-2 mutational changes are currently mainly used to observe the geographical transmission of the infection. As long as the virus strains do not differ in virulence, transmission capacity or stability, it will not be of epidemiological significance. The currently spreading D614G mutation is believed to favor more efficient virus transmission, but it does not mean a more severe disease (Callaway 2020).

The evolution of a virus is due to environmental pressure. In the event of the COVID-19 pandemic, the percentage of sensitive people is of primary importance. It is difficult to imagine a selection pressure for SARS-CoV-2 when people who are insensitive as a result of passing the infection and acquiring immunity (even short-term, for 1–2 years) do not constitute more than 50% of the population, which could slow down the spread of the virus.

In the event of influenza virus outbreaks, antigenically distinct virus strains cause larger outbreaks every few years when previous strains can no longer find susceptible hosts. However, coronaviruses, unlike the flu virus, do not appear to induce durable immunity. Thus, there may not be the selection pressure described above on the virus, and therefore the virus may not change significantly and in its current or similar form will become the fifth human commonly occurring coronavirus forever.

## How the Pandemic will Develop

The initial development of the pandemic, related to the spread of an airborne- or droplet-transmitted virus in the world, broadly corresponds to the course of the influenza pandemic in the twentieth and twenty-first centuries (Spanish Influenza in 1918–1920, Asian Influenza in 1957, Hong Kong Influenza in 1968, “Swine” Influenza in 2009) and different predicted pandemic models. However, the further development of the current pandemic may differ from an influenza pandemic due to differences in genetic and antigenic properties between orthomyxoviruses and coronaviruses. The most important factors are the lower genetic variability of coronaviruses and the likely short-term duration of immunity following SARS-CoV-2 infection.

Projected models for the future course of the pandemic were presented by Kissler et al. (2020). The authors predict that the current pandemic will last until 2022, and then the next wave of cases will occur after a two-year hiatus. Various models of the course of the pandemic were presented by Scudellari (2020), taking into account such factors as the longevity of post-infectious immunity and cross-immunity with other coronaviruses. These and any other predictions will of course be more accurate as more is known about the virus.

However, the course of the pandemic could be quite different if the following current assumptions prove to be incorrect: (1) SARS-CoV-2 is a new human virus that has permanently entered the list of human viruses. (2) Post-infection immunity is relatively short-lived. By analogy with known human common cold coronaviruses, it may not exceed 2–3 years. (3) The virus will not be under much pressure and there will be no significant mutational changes in the short term. This will distinguish it from influenza viruses, and it will resemble the situation with other coronaviruses and e.g. paramyxoviruses or picornaviruses.

Analyzing the pandemic's course to date and our knowledge about the virus, it can be expected that in the future, after the situation stabilizes, we will pass primary infection at a young age as a usually mild childhood disease. Later in life, cases of primary infection will pose a similar problem, as is the case of SARS-CoV-2 infection today. One analogy arises with primary adult infections with varicella, measles or mumps viruses. With the extinction or weakening of specific immunity after primary infection, multiple reinfections may occur. However, in such a case some partially preserved immunity may prevent severe complications. As a result, in a dozen or so years, SARS-CoV-2 infection will not pose such a threat as at present. Perhaps the natural history of the known common cold coronaviruses was similar, having an initial period of significant mortality. It was proposed that HCoV-OC43 entered humans around 1890 as the zoonotic transmission of bovine coronavirus resulting in pandemic (Vijgen et al. 2005). However, this hypothesis is difficult to prove, and the 1889–1890 pandemic is widely believed to have been caused by the H2N2 influenza virus.

Only vaccine development and mass prophylactic vaccinations can change created by different authors, but quite compatible scenarios.

It is important to realize that the current pandemic does not mean, of course, that there will not be another pandemic for another new pathogen in the future. And there is no reason why it might not happen soon.
